# Discharge diagnoses versus medical record review in the identification of community-acquired sepsis

**DOI:** 10.1186/s13054-015-0771-6

**Published:** 2015-02-16

**Authors:** Henry E Wang, Dylan R Addis, John P Donnelly, Nathan I Shapiro, Russell L Griffin, Monika M Safford, John W Baddley

**Affiliations:** Department of Emergency Medicine, University of Alabama School of Medicine, 619 19th Street South, OHB 251, Birmingham, AL 35249 USA; University of Alabama School of Medicine, Birmingham, Alabama USA; Department of Epidemiology, School of Public Health, University of Alabama at Birmingham, Birmingham, Alabama USA; Department of Medicine, Division of Preventive Medicine, University of Alabama School of Medicine, Birmingham, Alabama USA; Department of Emergency Medicine, Beth Israel Deaconess Medical Center, Boston, Massachusetts USA; Department of Medicine, Division of Infectious Diseases, University of Alabama School of Medicine, Birmingham, Alabama USA

## Abstract

**Introduction:**

We evaluated the accuracy of hospital discharge diagnoses in the identification of community-acquired sepsis and severe sepsis.

**Methods:**

We reviewed 379 serious infection hospitalizations from 2003 to 2012 from the national population-based reasons for geographic and racial differences in stroke (REGARDS) cohort. Through manual review of medical records, we defined criterion-standard community-acquired sepsis events as the presence of a serious infection on hospital presentation with ≥2 systemic inflammatory response syndrome criteria. We also defined criterion-standard community-acquired severe sepsis events as sepsis with >1 sequential organ failure assessment organ dysfunction. For the same hospitalizations, we identified sepsis and severe sepsis events indicated by Martin *et al.* and Angus *et al.* International Classifications of Diseases 9th edition discharge diagnoses. We evaluated the diagnostic accuracy of the Martin and Angus criteria for detecting criterion-standard community-acquired sepsis and severe sepsis events.

**Results:**

Among the 379 hospitalizations, there were 156 community-acquired sepsis and 122 community-acquired severe sepsis events. Discharge diagnoses identified 55 Martin-sepsis and 89 Angus-severe sepsis events. The accuracy of Martin-sepsis criteria for detecting community-acquired sepsis were: sensitivity 27.6%; specificity 94.6%; positive predictive value (PPV) 78.2%; negative predictive value (NPV) 65.1%. The accuracy of the Angus-severe sepsis criteria for detecting community-acquired severe sepsis were: sensitivity 42.6%; specificity 86.0%; PPV 58.4%; NPV 75.9%. Mortality was higher for Martin-sepsis than community-acquired sepsis (25.5% versus 10.3%, *P* = 0.006), as well as for Angus-severe sepsis than community-acquired severe sepsis (25.5 versus 11.5%, *P* = 0.002). Other baseline characteristics were similar between sepsis groups.

**Conclusions:**

Hospital discharge diagnoses show good specificity but poor sensitivity for detecting community-acquired sepsis and severe sepsis. While sharing similar baseline subject characteristics as cases identified by hospital record review, discharge diagnoses selected for higher mortality sepsis and severe sepsis cohorts. The epidemiology of a sepsis population may vary with the methods used for sepsis event identification.

**Electronic supplementary material:**

The online version of this article (doi:10.1186/s13054-015-0771-6) contains supplementary material, which is available to authorized users.

## Introduction

Sepsis is a major public health problem. Prior studies estimate that severe sepsis is responsible for over 750,000 hospital admissions, 570,000 emergency department visits, 200,000 hospital deaths and $16 billion in hospital expenditures in the United States annually [1,2].

An important step in reducing the national impact of sepsis is to quantify and characterize the affected patient population. Prior epidemiologic studies have applied a range of strategies to identify sepsis and severe sepsis using hospital diagnoses [1-8]. For example, Martin and colleagues identified sepsis hospitalizations using the International Classifications of Diseases, 9th edition (ICD-9) discharge diagnoses specific for sepsis or septicemia [3]. Angus and colleagues identified severe sepsis cases as hospitalizations with discharge diagnoses for both a serious infection and organ dysfunction [1].

While the analysis of administrative datasets leverages the efficiency of large pre-existing data, these approaches are limited by variations in physician documentation and hospital coding practices, and by the absence of physiologic or laboratory values [9]. Most importantly, discharge diagnoses are unable to distinguish initial community-acquired sepsis from later hospital-acquired sepsis. This distinction is important because sepsis detection and treatment strategies and the characteristics of affected patients probably differ between the two settings. Sepsis care guidelines generally focus on the early detection and treatment of community-acquired sepsis in the emergency department [10].

A more definitive strategy for identifying community-acquired sepsis is through the structured manual review of medical records, integrating information from physician and nursing notes, physiologic measurements and laboratory values during the patient’s initial hospital presentation. In this study we sought to determine the accuracy of discharge diagnoses for detecting community-acquired sepsis and severe sepsis among individuals hospitalized with a serious infection. We also sought to compare and contrast the characteristics of sepsis and severe sepsis cohorts identified by these methods.

## Materials and methods

### Study design

This study utilized data from the Reasons for Geographic and Racial Differences in Stroke (REGARDS) study, a national, population-based, longitudinal cohort, and REGARDS-Sepsis, an ancillary study identifying community-acquired sepsis events in the REGARDS cohort. The Institutional Review Board of the University of Alabama at Birmingham approved the study. Individuals provided written consent for participation in the REGARDS study.

### The REGARDS cohort

Designed to evaluate reasons for geographic and racial variations in stroke mortality, the REGARDS cohort is one of the largest ongoing national cohorts of community-dwelling individuals in the United Sates [11]. The cohort encompasses 30,239 individuals ≥45 years old, with 42% African American and 45% male. Approximately 69% of individuals are >60 years old. The REGARDS cohort includes individuals from all regions of the continental United States, with 21% of the cohort originating from the coastal plains of North Carolina, South Carolina and Georgia (the buckle of the stroke belt) and 35% originating from the remainder of North Carolina, South Carolina and Georgia plus Tennessee, Mississippi, Alabama, Louisiana and Arkansas (the stroke belt). The REGARDS cohort does not include Hispanics, where stroke mortality disparities are small to nonexistent.

The REGARDS study enrolled participants during 2003 to 2007. The REGARDS study determined baseline data for each participant through telephone interviews and in-person evaluations. Baseline information included medical history, functional status, health behaviors, physical characteristics (height, weight), physiologic measures (blood pressure, pulse, electrocardiogram) and an inventory of medications. The study obtained blood and urine specimens from each participant. Participants also completed self-administered questionnaires regarding diet, family history of diseases, psychosocial factors and prior residences.

As is customary for studies of this design, the REGARDS study contacts each participant on a semi-annual basis to determine the date, location and attributed reason for all emergency department visits and hospitalizations during the follow-up interval. For each participant death, the study reviews related death and medical records and interviews proxies to ascertain the circumstances of the death event.

### Selection of participants – identification of serious infection hospitalization events

We reviewed medical records for hospitalizations attributed by participants to a serious infection. We defined a serious infection using the taxonomy of Angus and colleagues [1] Two trained abstractors independently reviewed all pertinent medical records to confirm, first, the presence of a serious infection on initial hospital presentation and, second, its role as a major reason for hospitalization. The medical record review included physician and nursing clinical notes as well as laboratory records. Because some patients may not manifest sepsis signs until after initial emergency department presentation, we used the worst vital signs and laboratory values from first 28 hours of hospitalization, a period designed to encompass the emergency department and up to 1 day of inpatient care. The abstractors adjudicated discordances, with additional physician-level review as needed. Initial review indicated excellent inter-rater agreement for the presence of a serious infection (κ = 0.92). We examined hospitalization events occurring during 2003 to 2012.

The larger REGARDS-Sepsis study encompassed 3,431 serious infection hospitalizations observed among cohort participants. The current analysis focused on a subset of 379 serious infection events with ICD-9 discharge diagnoses available for review. This subset was identified during the early pilot stages of the larger REGARDS-Sepsis study.

### Identification of criterion-standard community-acquired sepsis and severe sepsis events

Among the serious infection events, we reviewed available hospital records to determine the criterion-standard presence of community-acquired sepsis and severe sepsis. We defined criterion-standard community-acquired sepsis as presentation to the hospital with an infection plus two or more systemic inflammatory response syndrome criteria, including: heart rate >90 beats/minute; fever (temperature >38.3°C or <36°C); tachypnea (>20 breaths/minute) or PCO_2_ < 32 mmHg; and leukocytosis (white blood cells >12,000 or <4,000 cells/mm^3^ or >10% band forms). Because of our focus on community-acquired sepsis, we used the most abnormal vital signs and laboratory test results for the initial 28 hours of hospitalization. We defined criterion-standard community-acquired severe sepsis as the presence of sepsis with concurrent organ dysfunction. We determined organ dysfunctions using Sequential Organ Failure Assessment (SOFA) based upon the most abnormal laboratory and physiologic findings during the first 28 hours of hospitalization for respiratory, renal, hepatic, cardiovascular, hematologic, and neurologic systems [12]. Missing values were coded as zero or absent in the determination of systemic inflammatory response syndrome and SOFA elements.

### Identification of sepsis and severe sepsis using discharge diagnoses

For the discharge diagnosis-based identification of sepsis, we used the ICD-9 codes of Martin and colleagues (Martin-sepsis) [3] (Additional file 1). We expanded the Martin-sepsis codes to include ICD-9 codes 785.52 (septic shock), 995.91 (sepsis) and 995.92 (severe sepsis) [3]. For the discharge diagnosis-based identification of severe sepsis, we used the ICD-9 codes of Angus and colleagues (Angus-severe sepsis), defining severe sepsis as the presence of discharge diagnoses for both an infection and organ dysfunction [1] (Additional file 2). As with prior studies, we expanded the Angus-severe sepsis criteria to include mechanical ventilation (ICD-9p 96.7) as a form of respiratory organ dysfunction [2]. If the diagnosis 785.52 (septic shock) or the diagnosis 995.92 (severe sepsis) was present, we classified the event as Angus-severe sepsis.

### Participant characteristics, hospital course and outcomes

The REGARDS participant sociodemographic data used in this analysis included age, sex, race, annual household income and education (years of school). The REGARDS-defined alcohol use categories included none, moderate (one drink per day for women or two drinks per day for men) and heavy (>1 drink per day for women and >2 drinks per day for men) [13]. Tobacco use categories included none, current and prior smoking.

Participant chronic medical conditions included atrial fibrillation, cancer history, chronic lung disease, chronic kidney disease, coronary artery disease, deep vein thrombosis, diabetes, dyslipidemia, hypertension, myocardial infarction, obesity, peripheral artery disease and stroke. Atrial fibrillation was identified by participant self-report or baseline electrocardiogram. Chronic kidney disease was defined as an estimated glomerular filtration rate <60 ml/minute/1.73 m^2^ based upon the CKD-EPI equation [14]. Coronary artery disease included a history of myocardial infarction or coronary intervention. Diabetes was defined as fasting glucose ≥126 mg/l (or glucose ≥200 mg/l for those not fasting) or the use of insulin or oral hypoglycemic agents. Dyslipidemia consisted of individuals with self-reported high cholesterol or the use of lipid-lowering medications. Hypertension included systolic blood pressure ≥140 mmHg, diastolic blood pressure ≥90 mmHg, or the self-reported use of antihypertensive agents. Myocardial infarction included those with a self-reported history of myocardial infarction or baseline electrocardiographic evidence of myocardial infarction.

Obesity was defined as a waist circumference >102 cm for males or >88 cm for females, or body mass index ≥30 mg/cm^2^ [15]. Participants self-reported the prior history of stroke (including transient ischemic attacks) or deep vein thrombosis. Peripheral artery disease included a history of lower extremity arterial bypass or leg amputation. The REGARDS study did not collect information on pulmonary conditions such as asthma and chronic obstructive pulmonary disease, and thus we defined chronic lung disease as participant use of pulmonary medications such as beta agonists, leukotriene inhibitors, inhaled corticosteroids, combination inhalers, ipatropium, cromolyn, aminophylline and theophylline.

Examined hospital course characteristics included the SOFA score for the first 28 hours of hospitalization, the Mortality in Emergency Department Sepsis (MEDS) score, admission to the ICU and hospital death [12,16,17].

### Statistical analysis

We assessed the accuracy of the Martin-sepsis criteria for detecting community-acquired sepsis events. We similarly determined the accuracy of the Angus-severe sepsis criteria for detecting community-acquired severe sepsis events. Examined diagnostic parameters included sensitivity, specificity, positive predictive value, negative predictive value, positive and negative likelihood ratio tests, and area under the receiver-operating characteristics curve.

To contrast participant characteristics and hospital course of Martin-sepsis with community-acquired subgroups, we followed the methodology of Muntner and colleagues, comparing the prevalence of each variable in events with discordant sepsis classifications (that is, Martin-sepsis[+]/community-acquired sepsis[−] or Martin-sepsis[−]/community-acquired sepsis[+]) [18]. We conducted the latter comparisons using univariable logistic regression, fitting a series of models with the discordance pattern as the dependent variable and each participant or hospital course characteristic as an independent variable. We made similar comparisons between Angus-severe sepsis and community-acquired severe sepsis cases. We conducted all statistical analyses using Stata version 12.1 (Stata, Inc., College Station, TX, USA).

## Results

The study included 379 hospitalizations with available discharge diagnoses. Among these cases, there were 156 (41.2%) criterion-standard community-acquired sepsis events (Table 1). Discharge diagnoses indicated 55 (14.5%) cases meeting Martin-sepsis criteria. The Martin-sepsis criteria detected 43 (27.6%) of 156 community-acquired sepsis cases (Table 2). There were 125 (33.0%) discordances between community-acquired sepsis and Martin-sepsis classifications.

**Table 1:** **Detection of community-acquired sepsis by Martin and colleagues’ discharge diagnoses** [3]

	**Community-acquired sepsis**		
**Sepsis identified by Martin and colleagues’ discharge diagnosis codes**	**Sepsis**	**No sepsis**	**Total**
Sepsis	43	12	55
No sepsis	113	211	324
Total	156	214	379

Total of 379 serious infection hospitalizations.

**Table 2:** **Classification accuracy of Martin and colleagues’ criteria in detecting community-acquired sepsis events, stratified by infection category** [3 **]**

**Diagnostic property**	**Lung ** **(** ***n*** **= 166)**	**Kidney ** **(** ***n*** **= 59)**	**Abdominal ** **(** ***n*** **= 73)**	**Skin ** **(** ***n*** **= 49)**	**Other ** **(** ***n*** **= 32)**	**Overall ** **(** ***n*** **= 379)**
Sensitivity (%)	18.8 (10.9 to 29.0)	44.4 (25.5 to 64.7)	4.4 (0.11 to 21.9)	40.0 (12.2 to 73.8)	68.8 (41.3 to 89.0)	27.6 (20.7 to 35.3)
Specificity (%)	98.8 (93.7 to 100.0)	90.6 (75.0 to 98.0)	98.0 (89.4 to 99.9)	94.9 (82.7 to 99.4)	68.8 (41.3 to 89.0)	94.6 (90.8 to 97.2)
Positive predictive value (%)	93.8 (69.8 to 99.8)	80.0 (51.9 to 95.7)	50.0 (1.3 to 98.7)	66.7 (22.3 to 95.7)	68.8 (41.3 to 89.0)	78.2 (65.0 to 88.2)
Negative predictive value (%)	56.7 (48.3 to 64.7)	65.9 (50.1 to 79.5)	69.0 (56.9 to 79.5)	86.0 (72.1 to 94.7)	68.8 (41.3 to 89.0)	65.1 (59.7 to 70.3)
Likelihood ratio positive	16.1 (2.2 to 119.0)	4.7 (1.5 to 15.1)	2.2 (0.1 to 33.2)	7.8 (1.7 to 36.7)	2.2 (0.99 to 9.9)	5.1 (2.8 to 9.4)
Likelihood ratio negative	0.82 (0.74 to 0.92)	0.61 (0.43 to 0.88)	0.98 (0.89 to 1.07)	0.63 (0.38 to 1.05)	0.46 (0.21 to 1.01)	0.77 (0.69 to 0.85)
Area under ROC curve	0.59 (0.54 to 0.63)	0.68 (0.57 to 0.78)	0.51 (0.47 to 0.56)	0.67 (0.51 to 0.84)	0.69 (0.41 to 0.89)	0.61 (0.57 to 0.65)

Data presented as mean (95% confidence interval). ROC, receiver operating characteristics.

While the sensitivity of the Martin-sepsis criteria for community-acquired sepsis events was low (27.6%), specificity was high (94.6%) (Table 2). Of the 113 community-acquired sepsis cases missed by the Martin-sepsis criteria, the infection types were as follows: lung (*n* = 65, 57.5%), kidney (*n* = 15, 13.3%), abdominal (*n* = 22, 19.5%), skin (*n* = 6, 5.3%) and other (*n* = 5, 4.4%). When stratified by infection type, sensitivity of the Martin-sepsis criteria was slightly higher for other infections (68.8%). Specificity was very high for lung (98.8%) and abdominal (98.0%) infections.

Compared with community-acquired sepsis, Martin-sepsis individuals were more likely to have atrial fibrillation and diabetes. (Table 3). Sociodemographics, health behaviors, other chronic medical conditions, initial SOFA and MEDS scores, and ICU admission rates were similar between community-acquired sepsis and Martin-sepsis cases. Hospital mortality was higher for Martin-sepsis than for community-acquired sepsis (25.5 vs. 10.3%, *P* = 0.006).

**Table 3:** **Baseline characteristics and hospital course of community-acquired sepsis events (identified by manual chart review) and sepsis events identified by Martin and colleagues’ discharge diagnoses**

**Characteristic**	**Community-acquired sepsis ** **(column %) ** **(** ***n*** **= 156)**	**Martin-sepsis (column%) ** **(** ***n*** **= 55)**	**Discordances favoring community-acquired sepsis (column %) ** **(** ***n*** **= 113)**	**Discordances favoring Martin-sepsis (column %) ** **(** ***n*** **= 12)**	***P*** **value ** **Community-acquired versus Martin-sepsis discordances** ^**a**^
Mean age (SD)	69.7 (9.1)	70.6 (8.8)	69.3 (9.3)	70.3 (10.3)	0.72
Sex					
Male	58.3	67.3	54.9	66.7	0.43
Female	41.7	32.7	45.1	33.3	
Race					
White	64.7	58.2	68.1	33.3	0.92
Black	35.3	41.8	31.9	66.7	
Income					
< $20,000	25.6	27.3	25.7	33.3	0.70
$20,000 to 34,000	37.2	41.8	34.5	33.3	
$35,000 to 74,000	21.8	21.8	21.2	16.7	
≥ $75,000	9.6	1.8	12.4	0.0	
Unknown	5.8	7.3	6.2	16.7	
Education					
Less than high school	22.4	21.8	21.2	8.3	0.70
High school graduate	31.4	30.9	32.7	41.7	
Some college	28.2	27.3	29.2	33.3	
College or higher	18.0	20.0	16.7	16.7	
Health behaviors					
Tobacco use					
Never	32.3	34.6	32.1	41.7	0.79
Past	52.3	52.7	50.9	41.7	
Current	15.5	12.7	17.0	16.7	
Alcohol use					
None	67.7	67.3	69.6	83.3	0.36
Moderate	29.0	29.1	27.7	16.7	
Heavy	3.2	3.6	2.7	0.0	
Chronic medical conditions					
Atrial fibrillation	15.6	17.3	17.8	50.0	0.03
Cancer	19.5	22.2	19.0	27.3	0.53
Chronic kidney disease	26.9	27.3	25.7	16.7	0.48
Chronic lung disease	18.0	7.3	22.1	8.3	0.22
Coronary artery disease	29.6	24.5	33.3	41.7	0.57
Deep vein thrombosis	12.9	16.4	11.6	16.7	0.62
Diabetes	35.3	47.3	34.5	83.3	<0.001
Dyslipidemia	66.7	70.0	64.2	55.6	0.61
Hypertension	70.3	35.5	71.4	58.3	0.36
Myocardial infarction	18.3	20.8	18.8	33.3	0.26
Obesity (abnormal BMI or WC)	62.8	63.6	61.1	50.0	0.46
Peripheral artery disease	3.9	5.5	3.54	8.33	0.47
Stroke	19.4	25.5	18.8	41.7	0.09
Infection type					
Lung	51.3	29.1	57.5	8.3	<0.001
Kidney	17.3	27.3	13.3	25.0	
Abdominal	14.7	3.6	19.5	8.3	
Skin	6.4	10.9	5.3	16.7	
Other	10.3	29.1	4.4	41.7	
28-hour SOFA score	2 (1 to 3)	2 (1 to 3)	1 (0 to 3)	2 (1 to 3)	0.56
Median (IQR) MEDS score	9 (6 to 13)	11 (6 to 13)	9 (6 to 11)	9 (6 to 10.5)	0.12
Admission to ICU	13.5	23.6	8.0	8.3	0.96
Hospital mortality	10.3	25.5	5.3	33.3	0.006

BMI, body mass index; IQR, interquartile range; MEDS, Mortality in Emergency Department Sepsis; SD, standard deviation; SOFA, Sequential Organ Failure Assessment; WC, waist circumference. ^a^The prevalence of each subject characteristic was evaluated by comparing the discordant pairs for each sepsis definition.

Among the 379 serious infection hospitalizations, there were 122 (32.2%) criterion-standard community-acquired severe sepsis events (Table 4). Discharge diagnoses indicated 89 (23.5%) cases meeting Angus-severe sepsis criteria. The Angus-severe sepsis criteria detected 52 (42.6%) of 122 community-acquired severe sepsis cases. While sensitivity of the Angus-severe sepsis criteria for community-acquired severe sepsis events was low (42.6%), specificity was higher (86.0%) (Table 5). Of the 70 community-acquired severe sepsis events missed by the Angus-severe sepsis criteria, the infection types were as follows: lung (*n* = 36, 51.4%), kidney (*n* = 13, 18.6%), abdominal (*n* = 12, 17.1%), skin (*n* = 4, 5.7%) and other (*n* = 5, 7.1%). When stratified by infection type, the sensitivity of Angus-severe sepsis criteria was slightly higher for other infections (58.3%), and specificity was slightly higher for skin (90.5%) and other infections (90.0%).

**Table 4:** **Detection of community-acquired severe sepsis by Angus and colleagues’ discharge diagnoses**

	**Community-acquired severe sepsis**		
**Severe sepsis identified by Angus and colleagues’ discharge diagnoses**	**Severe sepsis**	**No severe sepsis**	**Total**
Severe sepsis	52	37	89
No severe sepsis	70	220	290
Total	122	257	379

Total of 379 serious infection hospitalizations.

**Table 5:** **Diagnostic accuracy of Angus and colleagues’ criteria for detecting community-acquired severe sepsis events, stratified by infection category** [1 **]**

**Diagnostic property**	**Lung ** **(** ***n*** **= 166)**	**Kidney ** **(** ***n*** **= 59)**	**Abdominal ** **(** ***n*** **= 73)**	**Skin ** **(** ***n*** **= 49)**	**Other ** **(** ***n*** **= 32)**	**Overall ** **(** ***n*** **= 379)**
Sensitivity (%)	41.0 (28.6 to 54.3)	43.5 (23.2 to 65.5)	36.8 (16.3 to 61.6)	42.9 (9.9 to 81.6)	58.3 (27.7 to 84.8)	42.6 (33.7 to 51.9)
Specificity (%)	83.8 (75.3 to 90.3)	77.8 (60.8 to 89.9)	88.9 (77.4 to 95.8)	90.5 (77.4 to 97.3)	90.0 (68.3 to 98.8)	85.6 (80.7 to 88.7)
Positive predictive value (%)	59.5 (43.3 to 74.4)	55.6 (30.8 to 78.5)	53.8 (25.1 to 80.8)	42.9 (9.9 to 81.6)	77.8 (40.0 to 97.2)	58.4 (47.5 to 68.8)
Negative predictive value (%)	71.0 (62.1 to 78.8)	68.3 (51.9 to 81.9)	80.0 (67.7 to 89.2)	90.5 (77.4 to 97.3)	78.3 (56.3 to 92.5)	75.9 (70.5 to 80.7)
Likelihood ratio positive	2.5 (1.5 to 4.3)	2.0 (0.9 to 4.2)	3.3 (1.3 to 8.6)	4.5 (1.3 to 15.9)	5.8 (1.4 to 23.6)	3.0 (2.1 to 4.3)
Likelihood ratio negative	0.70 (0.56 to 0.88)	0.73 (0.49 to 1.08)	0.71 (0.50 to 1.01)	0.63 (0.33 to 1.21)	0.46 (0.23 to 0.92)	0.67 (0.57 to 0.79)
Area under ROC curve	0.62 (0.55 to 0.70)	0.61 (0.48 to 0.73)	0.63 (0.51 to 0.75)	0.67 (0.46 to 0.87)	0.74 (0.58 to 0.90)	0.64 (0.59 to 0.69)

Data presented as mean (95% confidence interval). ROC, receiver operating characteristics.

Compared with community-acquired severe sepsis, Angus-severe sepsis individuals were older and exhibited higher initial SOFA scores (Table 6). Other sociodemographics, health behaviors, chronic medical conditions, MEDS scores, and ICU admission rates were similar between community-acquired severe sepsis and Angus-severe sepsis cases. Hospital mortality was higher for Angus-severe sepsis than for community-acquired severe sepsis (22.5% vs. 11.5%, *P* = 0.002).

**Table 6:** **Baseline participant characteristics and hospital course of community-acquired severe sepsis events (identified by manual chart review) and severe sepsis events identified by Angus and colleagues’ discharge diagnoses**

**Characteristic**	**Community-acquired severe sepsis (column %) ** **(** ***n*** **= 122)**	**Angus-severe sepsis (column %) ** **(** ***n*** **= 89)**	**Discordances favoring community-acquired severe sepsis (column %) ** **(** ***n*** **= 70)**	**Discordances favoring Angus-severe sepsis (column %) ** **(** ***n*** **= 37)**	***P*** **value ** **Community-acquired versus Angus-severe sepsis discordances** ^**a**^
Age (mean ± SD)	70.3 (8.9)	72.1 (8.6)	69.3 (8.9)	72.9 (8.4)	0.049
Sex					
Male	65.9	65.2	62.9	64.9	0.84
Female	34.1	34.8	37.1	35.1	
Race					
White	65.6	68.5	70.0	18.9	0.21
B lack	34.4	31.5	30.0	81.1	
Income					
< $20,000	23.8	29.2	20.0	29.7	0.43
$20,000 to 34,000	36.9	36.0	38.6	37.8	
$35,000 to 74,000	21.3	20.2	18.6	13.5	
≥ $75,000	10.7	5.6	14.3	5.4	
Unknown	7.4	9.0	8.6	13.5	
Education					
Less than high school	23.8	22.5	22.9	18.9	0.91
High school graduate	27.1	28.9	31.4	37.8	
Some college	30.3	32.6	21.4	18.9	
College or higher	18.9	16.9	24.3	24.3	
Health behaviors					
Tobacco use					
Never	31.4	28.4	37.1	35.1	0.98
Past	53.7	55.7	50.0	51.4	
Current	14.9	15.9	12.9	13.5	
Alcohol use					
None	65.3	71.9	63.8	78.4	0.29
Moderate	30.6	24.7	31.9	18.9	
Heavy	4.1	3.4	4.4	2.7	
Chronic medical conditions					
Atrial fibrillation	16.7	18.4	17.1	21.6	0.58
Cancer	18.1	25.8	11.3	24.0	0.16
Chronic kidney disease	32.0	34.8	27.1	29.7	0.78
Chronic lung disease	12.3	13.5	12.9	16.2	0.64
Coronary artery disease	33.6	37.5	38.2	51.4	0.20
Deep vein thrombosis	14.9	13.6	13.0	8.3	0.46
Diabetes	34.4	42.1	32.9	50.0	0.09
Dyslipidemia	61.8	74.7	61.8	72.7	0.28
Hypertension	68.9	75.3	68.6	83.8	0.08
Myocardial infarction	21.7	22.7	26.1	32.4	0.49
Obesity (abnormal BMI or WC)	63.1	67.4	58.6	62.9	0.52
Peripheral artery disease	4.1	7.9	1.43	8.11	0.09
Stroke	20.7	20.2	18.8	16.2	0.74
Infection type					
Lung	50.0	47.2	51.4	46.0	0.88
Kidney	18.9	20.2	18.6	21.6	
Abdominal	15.6	14.6	17.1	16.2	
Skin	5.7	7.9	5.7	10.8	
Other	9.8	10.1	7.1	5.4	
28-hour SOFA score	2 (1 to 3)	3 (2 to 4)	2 (1 to 3)	3 (1 to 4)	0.01
Median (IQR) MEDS score	10.5 (6 to 13)	11 (8 to 14)	9 (6 to 11)	9 (8 to 14)	0.25
Admission to ICU	16.4	24.7	7.1	18.9	0.07
Hospital mortality	11.5	22.5	4.3	24.3	0.002

BMI, body mass index; IQR, interquartile range; MEDS, Mortality in Emergency Department Sepsis; SD, standard deviation; SOFA, Sequential Organ Failure Assessment; WC, waist circumference. ^a^The prevalence of each subject characteristic was evaluated by comparing the discordant pairs for each sepsis definition.

## Discussion

Prior studies have used hospital discharge diagnoses to characterize the epidemiology of sepsis and severe sepsis [1-7]. Using hospital event data from a national population-based cohort, our study offers unique perspectives of this strategy for sepsis identification. Our study confirms that the Martin-sepsis and Angus-severe sepsis diagnoses are specific but poorly sensitive for identifying community-acquired sepsis and severe sepsis determined through structured review of initial hospital data. Our study also confirmed that the Martin-sepsis and Angus-severe sepsis criteria select for populations with higher hospital mortality rates.

The findings of this study neither support nor refute the merits of any particular sepsis identification strategy. Rather, our analysis highlights the distinct features and trade-offs of each approach (Table 7). Advantages of medical record review include the use of physiologic measurements and laboratory test values, and better affirmation of a potential linkage with an underlying infection. However, manual chart review is clearly more arduous. While efficiently utilizing existing hospital data, discharge diagnoses may miss sepsis or severe sepsis cases not recognized or documented by clinicians, or not coded by billing personnel. The community-acquired sepsis and severe sepsis cases missed by the Martin-sepsis and Angus-severe sepsis criteria were predominantly due to lung infections, suggesting that gaps in sepsis coding and documentation practices may be most pronounced in this subset.

**Table 7:** **Comparison of manual medical record review for identification of criterion-standard community-acquired sepsis and severe sepsis, and of Martin and colleagues’ and Angus and colleagues’ discharge diagnoses for sepsis identification** [1 **,** 3 **]**

**Characteristic**	**Manual medical record review for identification of community-acquired sepsis and severe sepsis**	**Martin and colleagues’ and Angus and colleagues’ discharge diagnoses for identification of sepsis and severe sepsis**
Data source	Manual review of initial hospital records (emergency department and admission notes and laboratory test results from the first 28 hours of hospitalization)	Hospital discharge diagnosis codes
Criteria for sepsis or severe sepsis	Sepsis [infection + ≥2 SIRS criteria]	Sepsis: ICD-9 discharge diagnoses for sepsis (Martin and colleagues; Additional file 1)
	Severe sepsis [sepsis + ≥1 SOFA organ dysfunction]	Severe sepsis: ICD-9 discharge diagnoses for [infection + organ dysfunction] (Angus and colleagues; Additional file 2)
Timeframe/horizon	28 hours	Entire hospital stay
Strengths	Based upon structured review of medical records	Can utilize existing hospital discharge data
	Focused on initial hospitalization (community-acquired sepsis)	
	Verified connection between infection and sepsis (infection must be major reason for hospitalization)	
	Extensive data on pre-existing comorbid conditions	
Limitations	Limited to the REGARDS cohort	Limited information on pre-existing comorbid conditions
	Requires manual review of medical records	Cannot differentiate initial (community-acquired) from later (hospital-acquired) sepsis
	Limited to initial hospitalization presentation and records – cannot detect later (hospital-acquired) sepsis	Depends upon accuracy of coded discharge diagnoses
		Assumes connection between coded infection and organ dysfunction (Angus and colleagues)

ICD-9, International Classifications of Diseases, 9th edition; REGARDS, Reasons for Geographic and Racial Differences in Stroke; SIRS, systemic inflammatory response syndrome; SOFA, Sequential Organ Failure Assessment.

The Martin-sepsis and Angus-severe sepsis criteria are believed to be poorly sensitive but highly specific for identifying sepsis and severe sepsis hospitalizations. One would expect Martin and colleagues’ and Angus and colleagues’ criteria to exhibit increased sensitivity and decreased specificity when limited to community-acquired sepsis and severe sepsis detection. However, we observed that the low sensitivity and high specificity of these criteria persisted even when limited in this manner. This finding has two important implications. First, the low sensitivity may reflect inherent underdetection of all sepsis and severe sepsis events, not the relative proportions of community-acquired versus hospital-acquired sepsis cases. Secondly, the high specificity suggests that, when present, sepsis and severe sepsis-related discharge diagnoses may tend to be associated with community-acquired cases.

The varying approaches to sepsis identification may result in study populations with markedly different patient characteristics. Using hospital discharge data for Sweden, Wilhelms and colleagues showed threefold variation in the estimated incidence of severe sepsis using three different definitions for severe sepsis [19]. Similarly, using US national data, Gaieski and colleagues observed twofold variations in hospital severe sepsis mortality with differing severe sepsis definitions [7]. Using data on 1,735 patients from a single academic medical center, Whittaker and colleagues showed that that the Angus-severe sepsis discharge criteria tend to select for a more severely ill severe sepsis cohort, with increased lactates, rates of ICU admission, Acute Physiology and Chronic Health Evaluation II scores and 28-day mortality [5]. Our study affirmed higher rates of hospital mortality among Martin-sepsis and Angus-severe sepsis cases. Curiously, our differing rates of hospital mortality were accompanied by similarities in initial MEDS and SOFA scores, rates of ICU admission, and baseline comorbidity profiles. These observations may indicate that the Martin-sepsis and Angus-severe sepsis criteria select patients that tend to decompensate later in the hospital course.

Our study adds to the body of literature evaluating strategies for identifying sepsis and severe sepsis. Select studies have evaluated the accuracy of the Martin and colleagues’ and Angus and colleagues’ criteria for sepsis and severe sepsis detection. Using expert review of medical records, Iwashyna and colleagues evaluated the accuracy of Angus and colleagues’ criteria for severe sepsis identification, finding similarly low sensitivity (50%) but slightly higher specificity (96%) than in our series [9]. However, their series originated from a single center and was limited to a random selection of 111 hospitalizations. Poulose and colleagues conducted a similar analysis evaluating the accuracy of discharge diagnoses for identifying 99 cases of septic shock, but this study was limited to ICU patients [20]. Our contrasting analysis encompassed a larger series of sepsis and severe sepsis events from multiple centers, used structured laboratory and vital signs to define organ dysfunction, and focused upon initial hospital presentation. We additionally evaluated Martin and colleagues’ criteria for sepsis, which was not done in the other studies [3].

### Limitations

Because the REGARDS cohort contains individuals older than 45 years old only, we could not characterize sepsis in younger individuals. By design, the REGARDS cohort includes only African Americans and whites, and thus these results may not generalize to other ethnic groups. While the REGARDS trial was designed to study stroke, not sepsis, we were able to take advantage of important features of the study, including the large participant base, extensive baseline information and extended observation period. As is customary for studies of this design, the parent REGARDS study used participant reports of hospitalization events, and thus we may have underestimated the number of serious infection hospitalizations. However, our methodology encompassed detailed manual review of each reported event to verify pertinent clinical information. We note that recall and reporting bias are present in all of the strategies described in this study, including those based on discharge diagnoses.

As discussed previously, our study focused on a subset of 379 hospitalizations with hospital discharge data available at the time of analysis. We did not systematically sample events from the 3,431 serious infection hospitalizations of the larger REGARD-Sepsis study. Also, our analysis examined only hospitalizations attributed to an infection; unidentified sepsis events may have occurred among other hospitalizations. We are in the process of linking serious infection events with Medicare claims data, affording a more robust analysis that may address these concerns.

We applied the Martin-sepsis and Angus-severe sepsis criteria using all available discharge diagnoses. The observed specificities may be even higher if we were able to narrow the analysis to conditions present on hospital admission. However, present-on-admission flags are available only with select hospital discharge datasets [21]. To classify sepsis using hospital data we used information available from the first 28 hours of hospitalization – the observed number of sepsis and severe sepsis events (and potentially the sensitivity of Martin-sepsis and Angus-severe sepsis criteria) would have been higher with the availability of additional hospital data. Also, we coded missing values as zero or absent, which may have altered the number of criterion-standard sepsis and severe sepsis cases.

## Conclusion

Discharge diagnoses show good specificity but poor sensitivity for detecting community-acquired sepsis and severe sepsis events. Discharge diagnoses select for higher mortality sepsis and severe sepsis cohorts. The epidemiology of cohort may vary with differing approaches to sepsis event identification.

## Key messages

- The Martin-sepsis discharge diagnoses are poorly sensitive but highly specific for identifying community-acquired sepsis.
- The Angus-severe sepsis discharge diagnoses are poorly sensitive but highly specific for identifying community-acquired severe sepsis.
- The Martin-sepsis and Angus-severe sepsis criteria select for subgroups with higher mortality than those selected by medical record review. Other subject characteristics are similar.
- Epidemiologic studies of sepsis must consider the strengths and limitations of different sepsis detection strategies.

## Additional files

Additional file 1:
**Is a table presenting the ICD-9 discharge diagnoses for sepsis, adopted from Martin and colleagues [** 3 **].**
Additional file 2:
**Is a table presenting the ICD-9 infection and organ dysfunction discharge diagnoses, adopted from Angus and colleagues [** 1 **].** Severe sepsis defined by the presence of both infection and organ dysfunction.

## Abbreviations

ICD-9: International Classifications of Diseases, 9th edition
MEDS: Mortality in Emergency Department Sepsis
REGARDS: Reasons for Geographic and Racial Differences in Stroke
SOFA: Sequential Organ Failure Assessment
