# Supplementary material for: Discharge diagnoses versus medical record review in the identification of community-acquired sepsis
Source: Crit Care. 2015 Feb 16;19(1):42. doi: 10.1186/s13054-015-0771-6 (PMC4340494; doi:10.1186/s13054-015-0771-6)
Supplement: Additional file 1: — Is a table presenting the ICD-9 discharge diagnoses for sepsis, adopted from Martin and colleagues [ 3 ]. [file 13054_2015_771_MOESM1_ESM.docx]

# Additional file 1: ICD-9 discharge diagnoses for sepsis, adopted from Martin, et al. [3]

| **ICD-9-CM**  **Code** | **Description** |
| --- | --- |
|  |  |
| 038-0.38.9 | Septicemia |
| 020.0 | Septicemic |
| 790.7 | Bacteremia |
| 117.9 | Disseminated fungal infection |
| 112.5 | Disseminated candida infection |
| 112.81 | Disseminated fungal endocarditis |
| 995.91 | Sepsis |
| 995.92 | Severe sepsis |
|  |  |
