# Supplementary material for: Discharge diagnoses versus medical record review in the identification of community-acquired sepsis
Source: Crit Care. 2015 Feb 16;19(1):42. doi: 10.1186/s13054-015-0771-6 (PMC4340494; doi:10.1186/s13054-015-0771-6)
Supplement: Additional file 2: — Is a table presenting the ICD-9 infection and organ dysfunction discharge diagnoses, adopted from Angus and colleagues [ 1 ]. Severe sepsis defined by the presence of both infection and organ dysfunction. [file 13054_2015_771_MOESM2_ESM.docx]

# Additional file 2: ICD-9 infection and organ dysfunction discharge diagnoses, adopted from Angus, et al. [1] Severe sepsis defined by the presence of both infection and organ dysfunction.

INFECTIONS

| **Infection Category** | **ICD-9-CM Code** | **ICD-9-CM Code Description** |
| --- | --- | --- |
|  |  |  |
| Parasitic | 001 | Cholera |
|  | 002 | Typhoid/paratyphoid fever |
|  | 003 | Other salmonella infection |
|  | 004 | Shigellosis |
|  | 005 | Other food poisoning |
|  | 008 | Intestinal infections due to Escherichia coli |
|  | 008.1 | Intestinal infections due to Arizona group of paracolon bacillus |
|  | 008.2 | Intestinal infections due to Aerobacter aerogenes |
|  | 008.3 | Intestinal infections due to Proteus (mirabilis morganii) |
|  | 008.4 | Intestinal infections due to unspecified bacteria |
|  | 008.5 | Bacterial enteritis, unspecified |
|  | 009 | Ill-defined intestinal infection |
|  | 013 | CNS tuberculosis |
|  | 018 | Miliary tuberculosis |
|  | 020 | Plague |
|  | 021 | Tularemia |
|  | 022 | Anthrax |
|  | 023 | Brucellosis |
|  | 024 | Glanders |
|  | 025 | Melioidosis |
|  | 026 | Rat-bite fever |
|  | 027 | Other bacterial zoonoses |
|  | 032 | Diphtheria |
|  | 033 | Whooping cough |
|  | 034 | Streptococcal throat/scarlet fever |
|  | 035 | Erysipelas |
|  | 036 | Meningococcal infection |
|  | 037 | Tetanus |
|  | 038 | Septicemia |
|  | 039 | Actinomycotic infections |
|  | 040 | Other bacterial diseases |
|  | 041 | Bacterial infection in other diseases not specified |
|  | 098 | Gonococcal infections |
|  | 100 | Leptospirosis |
|  | 101 | Vincent’s angina |
|  | 112 | Candidiasis, of mouth |
|  | 112.4 | Candidiasis, of lung |
|  | 112.5 | Candidiasis, disseminated |
|  | 112.8 | Candidiasis, of other specified sites |
|  | 114 | Coccidioidomycosis |
|  | 115 | Histoplasmosis |
|  | 116 | Blastomycotic infection |
|  | 117 | Other mycoses |
|  | 118 | Opportunistic mycoses |
|  |  |  |
| Nervous | 320 | Bacterial meningitis |
|  | 321 | Cryptococcal meningitis |
|  | 321.1 | Meningitis in other fungal diseases |
|  | 324 | CNS abcess |
|  | 325 | Phlebitis of intracranial sinus |
|  | 360 | Purulent endophthalmitis |
|  | 376 | Acute inflammation of orbit |
|  | 380.14 | Malignant otitis externa |
|  | 383 | Acute mastoiditis |
|  |  |  |
| Circulatory | 420.99 | Acute pericarditis due to other specified organisms |
|  | 421 | Acute or subacute endocarditis |
|  |  |  |
| Respiratory | 461 | Acute sinusitis |
|  | 462 | Acute pharyngitis |
|  | 463 | Acute tonsillitis |
|  | 464 | Acute laryngitis/tracheitis |
|  | 465 | Acute upper respiratory infection of multiple sites/not otherwise specified |
|  | 475 | Peritonsillar abscess |
|  | 481 | Pneumococcal pneumonia |
|  | 482 | Other bacterial pneumonia |
|  | 485 | Bronchopneumonia with organism not otherwise specified |
|  | 486 | Pneumonia, organism not otherwise specified |
|  | 491.21 | Acute exacerbation of obstructive chronic bronchitis |
|  | 494 | Bronchiectasis |
|  | 510 | Empyema |
|  | 513 | Abscess of lung and mediastinum |
|  |  |  |
| Digestive | 522.5 | Periapical abscess without sinus |
|  | 522.7 | Periapical abscess with sinus |
|  | 526.4 | Inflammatory conditions of the jaw |
|  | 527.3 | Abscess of the salivary glands |
|  | 528.3 | Cellulitis and abscess of oral soft tissue |
|  | 540 | Acute appendicitis |
|  | 541 | Appendicitis not otherwise specified |
|  | 542 | Other appendicitis |
|  | 562.01 | Diverticulitis of the small intestine without hemorrhage |
|  | 562.03 | Diverticulitis of the small intestine with hemorrhage |
|  | 562.11 | Diverticulitis of colon without hemorrhage |
|  | 562.13 | Diverticulitis of colon with hemorrhage |
|  | 566 | Abscess of the anal and rectal regions |
|  | 567 | Peritonitis |
|  | 569.5 | Intestinal abscess |
|  | 569.61 | Infection of colostomy or enterostomy |
|  | 569.83 | Perforation of intestine |
|  | 572 | Abscess of liver |
|  | 572.1 | Portal pyemia |
|  | 575 | Acute cholecystitis |
|  |  |  |
| Genitourinary | 590 | Kidney infection |
|  | 599 | Urinary tract infection not otherwise specified |
|  | 601 | Prostatic inflammation |
|  | 604 | Orchitis and epididymitis |
|  | 614 | Female pelvic inflammation disease |
|  | 615 | Uterine inflammatory disease |
|  | 616.3 | Abscess of Bartholin’s gland |
|  | 616.4 | Other abscess of vulva |
|  |  |  |
| Pregnancy | 634 | Spontaneous abortion, complicated by genital tract and pelvic infection |
|  | 635 | Legally induced abortion, complicated by genital tract and pelvic infection |
|  | 636 | Illegally induced abortion, complicated by genital tract and pelvic infection |
|  | 637 | Unspecified abortion, complicated by genital tract and pelvic infection |
|  | 638 | Failed attempted abortion, complicated by genital tract and pelvic infection |
|  | 639 | Complications following abortion and ectopic and molar pregnancies |
|  | 646.6 | Infections of genitourinary tract in pregnancy |
|  | 658.4 | Infection of amniotic cavity |
|  | 670 | Major puerperal infection |
|  | 675.1 | Abscess of breast |
|  |  |  |
| Skin | 681 | Cellulitis, finger/toe |
|  | 682 | Other cellulitis or abscess |
|  | 683 | Acute lymphadenitis |
|  | 685 | Pilonidal cyst, with abscess |
|  | 686 | Other local skin infection |
| Musculoskeletal | 711 | Pyogenic arthritis |
|  | 728.86 | Necrotizing fasciitis |
|  | 730 | Osteomyelitis |
|  |  |  |
| Other | 790.7 | Bacteremia |
|  | 958.3 | Posttraumatic wound infection, not elsewhere classified |
|  | 996.6 | Infection or inflammation of device/graft |
|  | 998.5 | Postoperative infection |
|  | 999.3 | Infectious complication of medical care not otherwise classified |
|  |  |  |

*ORGAN DYSFUNCTION*

| **Organ System Category** | **ICD-9-CM Code** | **ICD-9-CM Code Description** |
| --- | --- | --- |
|  |  |  |
| Cardiovascular | 458 | Orthostatic hypotension |
|  | 458.8 | Other specified hypotension |
|  | 458.9 | Hypotension, unspecified |
|  | 785.5 | Shock without mention of trauma |
| Hematologic | 286.6 | Defibrination syndrome |
|  | 286.9 | Other and unspecified coagulation defects |
|  | 287.4 | Secondary thrombocytopenia |
|  | 287.5 | Thombocytopenia, unspecified |
| Hepatic | 570 | Acute and subacute necrosis of liver |
|  | 573.4 | Hepatic infarction |
| Neurologic | 293 | Transient organic psychosis |
|  | 348.1 | Anoxic brain damage |
|  | 348.3 | Encephalopathy |
| Renal | 584 | Acute renal failure |
| Respiratory | 518.8 | Respiratory failure |
|  | 786.03 | Apnea |
|  | 799.1 | Respiratory arrest |
|  |  |  |
